# Investigation of the effects of different denture base fabrication techniques and hard relining resin materials on the fixation of immediate provisional hybrid prosthesis to titanium cylinders

**DOI:** 10.1186/s12903-025-05862-1

**Published:** 2025-05-09

**Authors:** Burcu Kanat-Ertürk, Cansu Akarsu, Önjen Tak

**Affiliations:** 1 Department of Prosthodontics, Faculty of Dentistry, Kocaeli University, Kocaeli, Turkey; 2 Department of Prosthodontics, Faculty of Dentistry, Istanbul Aydin University, Istanbul, Turkey

**Keywords:** Provisional hybrid denture base materials, Immediate hybrid prosthesis, Hard relining resin materials, Milling, 3D printing

## Abstract

**Background:**

Denture base fabrication techniques and hard relining resins play critical roles in the clinical durability of implant supported immediate provisional hybrid prostheses (IPHPs). This study aimed to investigate the effects of different denture base fabrication techniques and hard relining resins on the fixation of IPHPs to titanium cylinders using a push-out test, and observe the failure types.

**Methods:**

A total of 140 denture base acrylic resin specimens (diameter: 24 mm, height: 4 mm) were fabricated using four techniques: milling, 3D printing, injection molding, and conventional heat-polymerization. Holes in 10 mm diameter were drilled at the center of each specimen using an industrial drill. Then, titanium cylinders (Opus Implant) were fixed to the specimens using five hard relining resin materials: acrylic resin-based (Ufi Gel Hard)(UGH), heat-polymerized acrylic resin (Futura Basic Hot)(FBH), autopolymerizing composite resin (Quick Up)(QP), autopolymerizing denture repair resin based on diacrylate (Qu-resin)(QR), and autopolymerizing low shrinkage modelling acrylic resin (Pattern resin LS)(PR) (*n* = 7). Following 5000 thermal-cycles, a push-out test was performed using a universal testing machine (Test Control Systems). Data were statistically analyzed with two-way analysis of variance (ANOVA) and Tukey post-hoc test (SPSS26, *p* =.05).

**Results:**

Denture base fabrication techniques, hard relining resin materials, and their interactions had significant effects on the push-out forces (*p* <.001). Statistically significant differences among fabrication techniques were observed only in the QR group (*p* <.05), where heat-polymerization technique had the highest push-out forces. Among relining materials, PR exhibited the highest values for milling technique (*p* <.05). For 3D printing, PR (*p* =.007) and QR (*p* =.029) showed significantly higher values than UGH. For injection molding, PR was superior to QP (*p* =.012) and UGH (*p* =.001). For heat-polymerization technique, QR, PR and QP exhibited the higher values (*p* <.05). The most common failure type was adhesive failure between titanium cylinders and relining resins (ADHES-ti).

**Conclusions:**

Denture base fabrication techniques and relining resin types had significant effects on the push-out force. Conventional heat-polymerization technique provided the most consistent performance, whereas milling and 3D printing required careful selection of relining materials. These results can inform clinical decisions to improve IPHP durability and reduce complications.

## Background

Edentulous patients increasingly expect immediate fixed dental solutions to ensure comfort, maintain uninterrupted social interactions, fulfill their aesthetic and functional needs, and enhance their quality of life. To meet these expectations, implant supported provisional and definitive hybrid prosthesis, which combine the benefits of fixed and removable prostheses, has begun to be widely used [1–4]. Implant supported immediate provisional hybrid prostheses (IPHPs), which are performed on the same day as implant surgery in cases with sufficient implant torque values, address the patients’ aesthetic, phonetic and functional needs during the osseointegration, healing and final prosthesis periods, ultimately enhancing patients’ oral health-related quality of life [5, 6].

In the conventional transforming technique of IPHPs, a complete denture base fabricated before the surgery is drilled according to implant and multi-unit abutment positions. The gaps between the denture base and titanium cylinders, which are screwed to the multi-unit abutments, are filled with composite or acrylic resin hard relining resin materials to fix IPHPs to titanium cylinders [7]. This process transforms the denture base into an implant supported IPHP. The most common clinical complication of conventional fabricated IPHPs is fractures at the titanium cylinder fixation areas [6, 8, 9]. Therefore, the choice of hard relining resin material is one of the critical factors on the fracture resistance and clinical success of IPHPs. However, no studies in the literature have investigated the effects of relining resin materials on the fixation of titanium cylinders to IPHPs.

Denture bases used for IPHPs can be fabricated using computer-aided design and computer-aided manufacturing (CAD-CAM) systems with either subtractive (milling) or additive (3D printing) techniques, as well as using conventional techniques like heat-polymerization and injection molding [10]. In milling, prepolymerized homogeneous acrylic resin blocks, which exhibit enhanced mechanical and physical properties due to high temperature and pressure polymerization, are used. The subtractive method offers several benefits such as fewer residual monomers, reduced shrinkage, better tissue adaptability, thinner fabrication, and archiving capability [11, 12]. However, it is limited in handling intricate designs [13]. Conversely, additive manufacturing reduces material waste and allows for intricate designs using scarce resources, but it has drawbacks in terms of surface roughness and mechanical properties [14, 15].

There are various studies in the literature on the bond strengths between denture bases fabricated by subtractive or additive techniques and different hard relining resin materials [7, 13, 16]. No consensus has been reached on the gold standard of denture base fabrication technique and relining resin material. Moreover, the fixation of the milled and/or 3D printed IPHPs to any implant components have not been investigated in the literature until now. Previous studies have only investigated the mechanical strengths of conventional heat-polymerized denture bases to components like ball attachments or locator housings [17–19]. The aims of this in-vitro study were to investigate the effects of different denture base fabrication techniques and hard relining resin materials on the fixation of titanium cylinders to IPHPs using a push-out test, and to observe the failure types. The null hypothesis of the study was that different denture base fabrication techniques and hard relining resin materials would not affect the push-out force for the fixation of IPHPs to titanium cylinders.

## Methods

A power analysis was performed by using the G-Power 3.1.9.4 software (Heinrich, Heine University, Dusseldorf, Germany) to determine the number of specimens, based on data from a reference article [7]. The analysis indicated that a minimum of six specimens was required (effect size: 2.07, power: 0.95), and the subgroup size was set as seven in this study.

A total of 140 acrylic resin specimens were fabricated using four different techniques: milling, 3D printing, conventional heat-polymerization and injection molding (*N* = 35). Each fabrication group was then randomly divided into five relining resin subgroups: acrylic resin-based hard relining material (Ufi Gel Hard; Voco GmbH, Cuxhaven, Germany) (UGH), heat-polymerized acrylic resin (Futura Basic Hot; Schütz Dental GmbH, Rosbach, Germany) (FBH), autopolymerizing composite resin (Quick Up; Voco GmbH, Cuxhaven, Germany) (QP), autopolymerizing denture repair resin based on diacrylate (Qu-resin; Bredent, Senden, Germany) (QR), and autopolymerizing low shrinkage modelling acrylic resin (Pattern resin LS; GC America, Alsip, IL, USA) (PR). The brand names, manufacturers, chemical compositions, and lot numbers of the denture base materials and hard relining resin materials used in this study are listed in Table 1.

**Table 1:** Denture base materials and hard relining resin materials used in the study

Brand	Type	Manufacturer	Chemical composition	Lot number
KeyMill High Impact	Denture base for milling	Keystone, Myerstown, ABD	Ethylene glycol dimethacrylate	07022ORG
On Dent Cyclone Denture	Photopolymerizable denture base for 3D printing	On Dent, Izmir, Turkey	2,2’-Ethylenedioxyethyl dimethacrylate, Silicon dioxide, Diphenyl (2,4,6-trimethylbenzoyl) phosphine oxide, Aliphatic difunctional methacrylate, Cristobalite flour, 2-Propenoic acid, reaction products with pentaerythritol	230,120
SR-IvoBase High Impact	Heat polymerized PMMA denture base for injection molding	Ivoclar Vivadent, Schaan, Liechtenstein	Powder: 88.6% co-polymer, 10% poly (methyl methacrylate), 0.9% benzoyl-peroxide, pigments < 0.5 Liquid: 88.4% methyl methacrylate, 5.6% dimethacrylate, 6% co-polymer	VT0765
Futura Basic Hot Powder/ Liquid	Heat polymerized PMMA denture base for both conventional heat-polymerization and relining resin material	Schütz Dental, GmbH, Rosbach, Germany	Powder: pearl polymer made of poly (methyl methacrylate), pigments, initiators Liquid: methyl methacrylate, diurethane dimethacrylate, initiators, stabilizers	2,019,015,685 / 2,020,004,714
Qu resin / Qu connector	Autopolymerizing denture repair resin based on diacrylate	Bredent, Senden, Germany	~ 61% Acrylates ~ 37% Fillers ~ 2% Initiators, stabilizers, pigment	526,446 / 520,799
Quick Up / Quick up adhesive	Autopolymerizing composite resin	VOCO GmbH, Cuxhaven, Germany	5-10% 1,6-hexanediylbismethacrylate 1-2.5% Catalyst ≤ 2.5% Bis-GMA ≤ 2.5% Benzoyl peroxide	2,216,483 / 2,220,451
Ufi Gel Hard / Ufi Gel Hard Adhesive	Acrylic resin-based hard relining material	VOCO GmbH, Cuxhaven, Germany	Benzoyl peroxide (catalyst), hydroxyethylmethacrylate and acetone (adhesive)	2,212,661 / 2,214,593
Pattern Resin LS Powder/ Liquid	Autopolymerizing low shrinkage modelling acrylic resin	GC America, Alsip, IL, USA	Powder: Poly(methylmethacrylate), Polyethylmethacrylate Dibenzoyl peroxide Liquid: Methylmethacrylate 2-Hydroxyethyl-Methacrylate	2,012,171 / 2,105,051

**Fig. 1:**
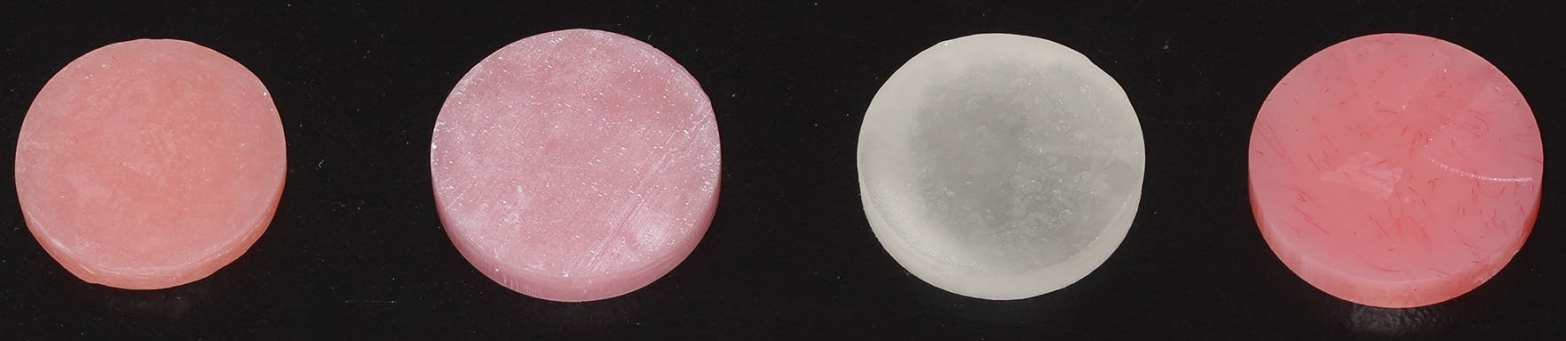
One representative specimen fabricated by injection molding, 3D printing, conventional heat-polymerization and milling technique, respectively

The specimens were designed in CAD software (Exocad 3.1; Rijeka, Darmstadt, Germany) in 24 mm diameter and in 4 mm height. For the milling technique, the designed discs were positioned within the denture base block in the software, connected to each other with sprues, and milled using a 5-axis milling unit (Coritec 250i; Imes-icore GmbH, Hessen, Germany) from a high-impact acrylic denture base disc (KeyMill™ Denture Base Disc, 25 mm; Keystone Industries, Gibbstown, New Jersey, USA). For the 3D printing technique, discs were fabricated additively with a digital light processing (DLP) 3D printer (Solflex 350^®^; Voco GmbH, Cuxhaven, Germany) using a photopolymerizable denture base (Cyclone Denture; On Dent, Izmir, Turkey). A layer thickness of 50 μm was used, and the discs were printed in horizontal orientation. The printed specimens were pre-cleaned for 3 min in a 99% isopropanol ultrasonic bath, followed by a 3-minute rinse in 99% isopropanol ultrasonic bath. Specimens were air-dried and post-polymerized using two cycles of UV-curing with flashlight (2000 flash for each side, totally 4000 flash) (Otoflash G171; NK-Optik, Baierbrunn, Germany) to ensure complete polymerization and mechanical stability. For injection molding and conventional heat-polymerization techniques, a milled disc served as a reference to standardize the specimens. In the injection molding technique, mixed resin material (SR IvoBase High Impact; Ivoclar Vivadent AG, Schaan, Liechtenstein) was packed into stone molds under 6 bar pressure (SR Ivocap; Ivoclar Vivadent AG) and polymerized in boiling water. For the conventional heat-polymerization technique, heat-polymerized acrylic resin (Futura Basic Hot; Schütz Dental GmbH) was packed into silicone molds formed by using the milled disc, and polymerized in a hot water bath. One representative specimen fabricated by each technique can be seen in Fig. 1.

**Fig. 2:**
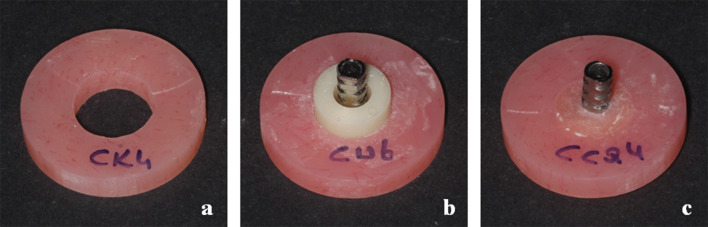
The process of specimen preparation, (**a**) representative specimen with a drilled gap of 10 mm in diameter, (**b**) space maintainer placed in the denture base to position the titanium cylinder, (**c**) fixation of the specimen to titanium cylinder by using hard relining resin material

For optimal properties of the hard relining resin, it is reported that at least 2 mm of acrylic resin is required for conventional hybrid prostheses, both from the cervical of the teeth to above the bar and from the base of the bar to the mandibular soft tissue [20, 21]. Therefore, in this study, 10 mm diameter holes were drilled at the center of the specimens to create 3 mm gaps around the 4 mm diameter titanium cylinders (Opus Implant; BA Dental, Istanbul, Turkey), allowing space for the resin materials and simulating the fixation of IPHPs to titanium cylinders (Fig. 2a). Drilling procedure was performed using an industrial drill that securely stabilized the specimens, minimizing vibration, preventing any positional shifts, and ensuring standardization. All drilling operations were carried out by the same operator, with the bur replaced after every five specimens. Due to the brittleness of the 3D printed acrylic resin, fractures occurred in half of the 3D printed specimens during the drilling process, requiring them to be refabricated. To ensure integrity, all specimens were examined under a microscope to confirm they were free of cracks. Finally, the drilled specimens were ultrasonically cleaned in distilled water for 5 min (BAKU steel ultrasonic cleaner BK-3550; Guangdong, China).

To standardize the placement of the titanium cylinders, a space maintainer (10 mm height, 10 mm outer diameter and 4 mm inner diameter) was designed in CAD software (Exocad 3.1; Rijeka) and milled from a PMMA acrylic disc (Tempo-CAD; On Dent) in the milling unit (Coritec 250i; Imes-icore GmbH). The titanium cylinders were positioned using the space maintainers, and were fixed in place with wax (Fig. 2b). Then, the space maintainers were removed, and the resulting gaps between titanium cylinders and denture bases were filled with five different hard relining resin materials, according to the manufacturers’ instructions (Fig. 2c) (*n* = 7). The resin materials used in the study and the application steps are provided in Table 2.

**Fig. 3:**
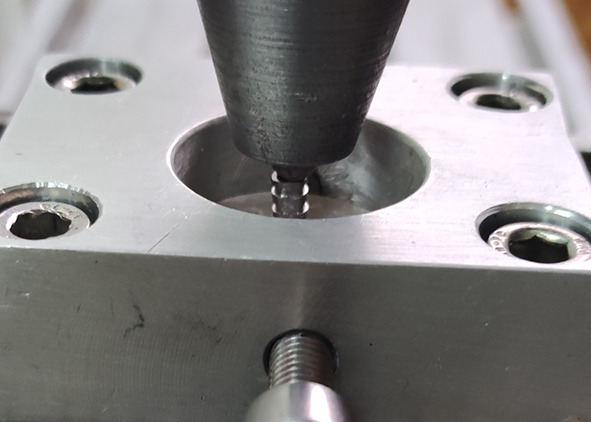
Push-out test aparatus

All specimens underwent 5000 thermal-cycles between 5ºC and 55ºC (SD Mechatronik Thermocycler; SD Mechatronik GmbH, Feldkirchen-Westerham, Germany) and a push-out test was conducted. To ensure proper detachment of the titanium cylinder, a 3 mm thick metal ring with a 20 mm diameter hole was placed under each specimen. A vertical push-out force was applied to the top of the titanium cylinders at a speed of 1 mm/min using a custom-made stainless steel ball with a diameter of 6 mm in a universal testing machine (TCS; Test Control Systems, Ankara, Turkey) (Fig. 3). The force was applied until the initial fracture occurred, and the corresponding values (N) (Mean ± SD) were recorded.

**Table 2:** Resin materials used in the study and application steps

Resin materials	Application steps
Qu resin / Qu connector (QR)	- The surface was sandblasted with 110 μm aluminum oxide. - The surface has been cleaned with oil-free compressed air. - Qu-connector implemented. Irradiated for 90 s. - Qu-resin was injected. - The polymerization time of the material is approx. 5 min.
Quick Up / Quick up adhesive (QP)	- Quick up adhesive was applied using an applicator. - Adhesive was air dried for 30 s. - Quick up was injected. - The polymerization time of the material is approx. 2 min.
Ufi Gel Hard / Ufi Gel Hard Adhesive (UGH)	- Conditioner was applied and left to dry for 30 s. - Ufi Gel hard powder/liquid was mixed homogeneously in a 3/1 ratio. - The polymerization time of the material is 7–8 min.
Pattern Resin LS Powder / Liquid (PR)	- The brush was wetted with the liquid and the powder was picked up, according to the manufacturer instruction. The gap was built up, layer by layer. - The working time of the material is 2–3 min. - The hardening time of the material is 4 min.
Futura Basic Hot Powder / Liquid (FBH)	- The mixing ratio of Futura Basic Hot is approximately 2.5 parts powder to 1 part liquid by weight. - After the powder of the material was added to the liquid, it was mixed for 30 s until a homogeneous mixture was obtained. - It was cooked in a pressure cooker at 95 degrees and 2–4 bar pressure for 20 min.

The Kolmogorov-Smirnov test was used to confirm that the obtained push-out force values followed a normal distribution. Afterwards, the main effects and interactions of the denture base fabrication techniques and relining resin materials were statistically analyzed using two-way analysis of variance (ANOVA) test in IBM SPSS 26 (SPSS; Chicago, IL, USA). Tukey post-hoc test was then performed for multiple comparison of the subgroups, with statistical significance set at *p* <.05.

The observed failures were categorized into five groups: 1- Cohesive in relining resin (COHES-res), 2- Cohesive in denture base (COHES-den), 3- Adhesive between titanium cylinder and relining resin (ADHES-ti), 4- Adhesive between relining resin and denture base (ADHES-den), 5- Mixed, indicating both adhesive and cohesive failures (MIX) [22].

## Results

According to the two-way ANOVA between-subjects effects test, both denture base fabrication techniques and hard relining resin materials statistically significantly affected the push-out force for the fixation of IPHPs to titanium cylinders on multi-unit abutments (*p* <.001). Moreover, denture base fabrication techniques*hard relining resin materials interaction was also statistically significant (*p* <.001), indicating that their combination influences the fixation of IPHPs to titanium cylinders.

The obtained push-out force values (N) (Mean ± SD) are presented in Table 3. Within the milling group, PR led to the highest push-out force values, followed by QP (*p* <.05). No statistically significant differences were observed among UGH, FBH, and QR (*p* >.05). In the 3D printing group, QR and PR demonstrated statistically significantly higher values than UGH (*p* <.05), whereas there was no statistically significant differences among FBH, QP, QR and PR. Similarly, no statistically significant differences were found among UGH, FBH, and QP. In the injection molding group, PR exhibited higher values compared to UGH (*p* =.001) and QP (*p* =.012), while no statistically significant differences were observed between PR and FBH or PR and QR (*p* >.05). Additionally, no statistically significant differences were observed between UGH and QP, or FBH and QR. In the heat-polymerization group, no statistically significant differences were observed among QR, PR and QP. However, QR and PR exhibited higher push-out force values than UGH (*p* <.001) and FBH (*p* =.002). QP also showed statistically significantly higher values than UGH (*p* =.002), but no statistically significant differences were found between QP and FBH or between FBH and UGH (*p* >.05).

For all hard relining resin groups, no statistically significant differences were observed among the denture base fabrication techniques, except for QR. QR showed the highest values in heat-polymerization group (*p* <.05) and the lowest in the milling group. Additionally, no statistically significant differences were found between the 3D printing and injection molding techniques for QR. Within CAD-CAM techniques, the 3D printing showed statistically significantly higher push-out force values than milling for QR. However, no statistically significant differences were observed between these two techniques for UGH, FBH, QP, and PR, although milling produced numerically higher values for PR, QP, and UGH (*p* >.05).

**Table 3:** Push-out force values (N) (Mean ± SD) for each group

	Milling	3D printing	Injection molding	Heat-polymerization
UGH	237 ± 85 ^a, A^	141 ± 33 ^a, b,A^	181 ± 79 ^a, b,A^	172 ± 62 ^a, A^
FBH	193 ± 41 ^a, A^	208 ± 89 ^a, A^	312 ± 139 ^a, A^	291 ± 48 ^a, b,A^
QP	348 ± 143^b, A^	250 ± 129 ^a, A^	242 ± 93 ^a, b,A^	381 ± 154 ^b, c,A^
QR	170 ± 59 ^a, A^	305 ± 98 ^a, c,B^	338 ± 132 ^a, B^	519 ± 34 ^c, C^
PR	471 ± 119 ^c, A^	335 ± 112 ^a, c,A^	454 ± 111 ^a, c,A^	501 ± 109 ^c, A^

Same superscript small letters represent no significant difference among five hard relining resins in the same column and same superscript capital letters indicate no significant difference among denture base fabrication techniques in the same row

**Fig. 4:**
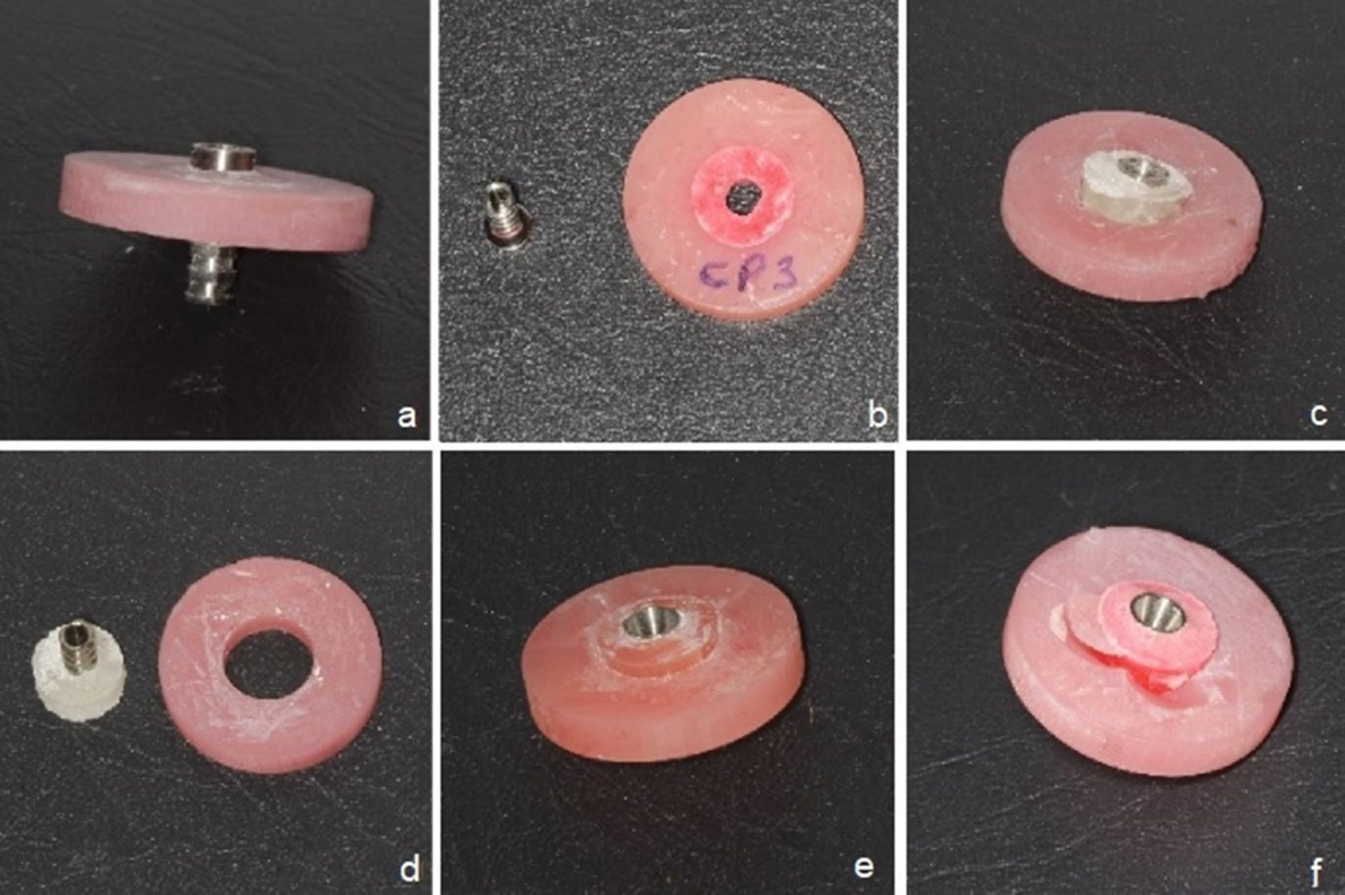
Failure types observed in the various resin material subgroups, (**a**-**b**) adhesive between titanium cylinder and relining resin (ADHES-ti), (**c**-**e**) adhesive between relining resin and denture base (ADHES-den), **(f**) mixed when both adhesive and cohesive failures were observed (MIX)

The distribution of failure types observed in each group is summarized in Table 4. Cohesive failures, both in relining resin (COHES-res) and denture base (COHES-den), were not observed in any group. Adhesive failure between the titanium cylinder and relining resin (ADHES-ti) was observed in all groups, except for UGH used in milling, injection molding, and heat-polymerization techniques, in addition to FBH and PR used in 3D printing technique. Adhesive failure between relining resin and denture base (ADHES-den) was observed for UGH in milling (*n* = 3), injection molding (*n* = 2), and heat polymerization (*n* = 2) techniques, respectively; and for FBH in 3D printing technique (*n* = 3). Mixed failure (MIX) was observed only in a single specimen fabricated with the 3D printing technique and bonded with PR. Representative failures for (ADHES-ti), (ADHES-den) and (MIX) are illustrated in Fig. 4a-f.

**Table 4:** The distribution of the failure types as “COHES-res/ COHES-den/ ADHES-ti/ ADHES-den/ MIX”

	Milling	3D printing	Injection molding	Heat-polymerization
UGH	0/0/4/3/0	0/0/7/0/0	0/0/5/2/0	0/0/5/2/0
FBH	0/0/7/0/0	0/0/4/3/0	0/0/7/0/0	0/0/7/0/0
QP	0/0/7/0/0	0/0/7/0/0	0/0/7/0/0	0/0/7/0/0
QR	0/0/7/0/0	0/0/7/0/0	0/0/7/0/0	0/0/7/0/0
PR	0/0/7/0/0	0/0/6/0/1	0/0/7/0/0	0/0/7/0/0

## Discussion

In this in-vitro study, push-out force values were investigated to analyze the fixation of implant supported IPHPs fabricated with different techniques, to titanium cylinders, by using various hard relining resin materials. The null hypothesis was rejected as statistically significant differences were observed in push-out force values among the denture base fabrication techniques for the QR group, and among the hard relining resin materials.

Among the hard relining resins used in this study, PR resulted in higher push-out force values, regardless of denture base fabrication techniques. This may be attributed to the application process of PR, which involves layering PMMA powder with a liquid-wetted brush layer by layer. Additionally, applying PMMA monomer to denture base improves bonding by increasing surface penetration with the aim of enhanced polymer interactions [23]. On the other hand, UGH exhibited numerically lower push-out force values in most fabrication technique groups. A previous study in the literature found that UGH exhibited the roughest surfaces around overdenture attachment housings, which could contribute to microcracks and bacterial build-up [19]. These factors may explain the lower push-out force values and the adhesive failures between UGH and denture base observed in our study. Water exposure during thermocycling may have further weakened the fixation of UGH to denture base.

High standard deviations for push-out force values were observed across all groups, which may have contributed to the lack of statistical significance in some comparisons. This variability could be potentially attributed to the macro-geometric design of the titanium cylinders. Although standardization was ensured through the design of specimens in CAD software, drilling by using an industrial drill to eliminate manual errors, microscopic examination to confirm the absence of cracks, fabrication under controlled conditions with detailed protocols, and verification of processes by an additional author, certain challenges remained. The non-fluid application of UGH, FBH, and PR may not have completely filled the gaps around the titanium cylinders, leading to variability. In turn, QP and QR, applied via injection, offered user-friendly application but may have introduced porosities during gap filling. Moreover, the application process for resin materials involves multiple critical stages that demand precision to prevent contamination or omissions, as well as accurate filling of the very small connection area with resin materials.

While no research has specifically examined the fixation of denture bases fabricated using different techniques to titanium cylinders with various resin materials, previous limited studies on the fixation of implant component housings to heat-polymerized denture bases provide some insights. Ozkır et al. [17] investigated the flexural strength of heat-polymerized denture base bonded to ball attachment housing by using different resin materials with 1 mm connection thickness. They found that heat-polymerized (Meliodent) and autopolymerized (Paladur) resins showed higher flexural strength than UGH and QP, with no statistically significant differences between heat-polymerized and autopolymerized resins or between UGH and QP [17]. Another study, which analyzed the push-out force values of heat-polymerized denture base bonded to locator housing by using different resin materials, reported that the highest values were obtained for heat-polymerized resin, with no statistically significant differences between autopolymerized (Triplex) and QP groups [18]. In our study, for the heat-polymerization fabrication technique, although PR showed numerically higher values than QP, there was no statistically significant difference, similar to the related studies [17, 18]. However, lower values were observed for heat-polymerized resin compared to other resins, in contrast with the aforementioned studies. Additionally, while numerically higher values were obtained for FBH than UGH, no statistically significant differences were observed. These discrepancies may stem from differences in the test methodologies, and macro-geometric designs of the implant components and specimens.

With the advent of CAD-CAM technologies, the flexural strength/push-out force values of milled and/or 3D printed denture bases bonded to the relining resin materials have become important areas of research for clinical applications. Panittaveekul et al. [7] compared the push-out force values of the milled denture base (Lucitone 199) bonded to the different resin materials, with and without thermocycling. In the thermocycled group, it was reported that PMMA acrylic resin groups (Jet denture base repair acrylic and Duralay) showed statistically significantly higher push-out force values than QP. The results of our study, in which PR showed statistically significantly higher push-out force values than QP for milling, is in accordance with the literature [7]. The higher values in the PMMA acrylic resin groups could be due to the chemical effects of methyl formate or methyl acetate, which is absent in the QP [7, 24]. The study’s failure type analysis showed that complete cohesive failures involving denture bases and resin material were observed in both PMMA acrylic resin groups, whereas partial fractures in resin material were seen in the QP [7]. In our study, adhesive failure between titanium cylinders and relining resin (ADHES-ti) was observed in all specimens for both PR and QP, except for a single specimen in the PR. Variations in test methodologies and the presence of the titanium cylinders may account for the observed differences in failure types.

Studies which compared the shear bond strengths of denture bases fabricated with different techniques to autopolymerized acrylic resin found that the lowest bond strengths were obtained for 3D printed denture bases, whereas no statistically significant differences were observed between the milled and conventional heat-polymerized denture bases [22, 25]. Similarly, Koseoglu et al. [13] reported that denture bases fabricated with 3D printing technique showed lower tensile bond strength values compared to conventional heat-polymerized denture bases when bonded to UGH. In our study, although numerically lower values were observed in 3D printing with respect to the milling and conventional heat-polymerization for both UGH and PR, no statistically significant differences were found among the three fabrication techniques. The partial difference in outcomes could be due to differences in specimen dimensions and relining materials, surface conditioning methods (monomer application, airborne particle abrasion and roughing with tungsten carbid bur) and absence/presence of implant components. Additionally, the lower bond strength of 3D printing compared to heat-polymerization technique could result from insufficient cross-linking of methyl methacrylate in 3D printed acrylic resins with the composition of bisphenol-A dimethacrylate [22, 26].

This study is the first to investigate the fixation of IPHPs fabricated with different techniques to titanium cylinders. The clinical implications of this research include that the fabrication techniques of denture base, the types and compositions of hard relining resins, and the clinician’s expertise in accurately performing this intricate process all significantly influence the durability of IPHPs during fixation to titanium cylinders and osseointegration process. Therefore, selecting the appropriate hard relining material, in accordance with the denture base’s fabrication technique and materials, is essential for maintaining the IPHPs stability and preventing complications, such as debonding or fractures. The outcomes of our study will assist to fill the deficiency of information on which combinations of denture base fabrication techniques and hard relining resin materials leads to higher push-out force values in the fixation of IPHPs to titanium cylinders. Moreover, achieving stable bonding through the selection of optimal materials and techniques may enhance the osseointegration process and improve patients’ quality of life by reducing the risk of failures and complications associated with IPHPs.

This study has several limitations that should be considered when interpreting the findings. The denture base specimens were fabricated in a simplified form that did not fully replicate the complex shape and curvature of the full-arch prosthesis used in clinical applications. This discrepancy may lead to differences in mechanical behavior between the tested specimens and actual prosthetic restorations. Additionally, in clinical application, the fixation of the titanium cylinder may not always occur at the center of the holes due to the insertion path of the full arch prosthesis. In contrast, this study maintained a uniform connection thickness around the titanium cylinder, which does not fully replicate the variability encountered in clinical practice. Although thermal cycling was applied to simulate intraoral conditions, mechanical aging was not performed in a chewing simulator. Since masticatory forces, micro-movements, and long-term wear could influence the stability of the relining resin materials, the push-out force values obtained in this study may not fully reflect the long-term clinical performance of IPHPs. Although power analysis was performed, the number of specimens in each group was relatively limited, which may have contributed to higher standard deviations. Given the lack of previous studies in the literature on this topic, this research can be considered as the pioneer study in the field and future studies are planned to obtain more comprehensive results. Additionally, the titanium cylinders’ length and macro-design may affect their retention within the denture base; however, this study did not compare titanium cylinders produced by different manufacturers. Future studies addressing these limitations could lead to more relevant findings clinically that may contribute to reducing IPHP-related complications and improving patients’ quality of life.

## Conclusions

In light of the findings of the current study, it can be concluded that the denture base fabrication techniques, hard relining resin materials, and their interactions, significantly affect the push-out force values of IPHPs fixed to titanium cylinders. Conventional heat-polymerization technique may be recommended for fabricating IPHPs, although milling and 3D printing CAD-CAM techniques may also be viable alternatives, provided that appropriate hard relining resin materials are selected to ensure adequate fixation. Different relining materials may lead to better results with various denture bases. Since the results vary depending on the combination of these materials, the relining resin material around the titanium cylinder may be selected to the specific denture base.

## Data Availability

No datasets were generated or analysed during the current study.

## Abbreviations

Fig: Figure
SD: Standard Deviation
CAD-CAM: Computer-aided design and computer-aided manufacturing
IPHPs: Immediate provisional hybrid prostheses
UGH: Ufi Gel Hard
FBH: Futura Basic Hot
QP: Quick Up
QR: Qu-resin
PR: Pattern resin LS
ANOVA: Analysis of variance
